# Percentage of Adolescents Meeting Federal Fruit and Vegetable Intake Recommendations — Youth Risk Behavior Surveillance System, United States, 2017

**DOI:** 10.15585/mmwr.mm7003a1

**Published:** 2021-01-22

**Authors:** Samantha J. Lange, Latetia V. Moore, Diane M. Harris, Caitlin L. Merlo, Seung Hee Lee, Zewditu Demissie, Deborah A. Galuska

**Affiliations:** ^1^Oak Ridge Institute for Science and Education, Oak Ridge, Tennessee; ^2^Division of Nutrition, Physical Activity, and Obesity, National Center for Chronic Disease Prevention and Health Promotion, CDC; ^3^Division of Population Health, National Center for Chronic Disease Prevention and Health Promotion, CDC; ^4^Division of Adolescent and School Health, National Center for HIV/AIDS, Viral Hepatitis, STD, and TB Prevention, CDC.

According to the 2020–2025 Dietary Guidelines for Americans, persons should consume fruits and vegetables as part of a healthy eating pattern to reduce their risk for diet-related chronic diseases, such as cardiovascular disease, type 2 diabetes, some cancers, and obesity.* A healthy diet is important for healthy growth in adolescence, especially because adolescent health behaviors might continue into adulthood (*1*). The U.S. Department of Agriculture (USDA) recommends minimum daily intake of 1.5 cups of fruit and 2.5 cups of vegetables for females aged 14–18 years and 2 cups of fruit and 3 cups of vegetables for males aged 14–18 years.^†^ Despite the benefits of fruit and vegetable consumption, few adolescents consume these recommended amounts (*2*–*4*). In 2013, only 8.5% of high school students met the recommendation for fruit consumption, and only 2.1% met the recommendation for vegetable consumption (*2*). To update the 2013 data, CDC analyzed data from the 2017 national and state Youth Risk Behavior Surveys (YRBSs) to describe the percentage of students who met intake recommendations, overall and by sex, school grade, and race/ethnicity. The median frequencies of fruit and vegetable consumption nationally were 0.9 and 1.1 times per day, respectively. Nationally, 7.1% of students met USDA intake recommendations for fruits (95% confidence interval [CI] = 4.0–10.3) and 2.0% for vegetables (upper 95% confidence limit = 7.9) using previously established scoring algorithms. State-specific estimates of the percentage of students meeting fruit intake recommendations ranged from 4.0% (Connecticut) to 9.3% (Louisiana), and the percentage meeting vegetable intake recommendations ranged from 0.6% (Kansas) to 3.7% (New Mexico). Additional efforts to expand the reach of existing school and community programs or to identify new effective strategies, such as social media approaches, might help address barriers and improve adolescent fruit and vegetable consumption.

The Youth Risk Behavior Surveillance System monitors prevalence of youth health behaviors that contribute to the leading causes of death and disability at the national, state, territorial, tribal, and large urban school district levels.^§^ Students complete anonymous, self-administered questionnaires during one class period. CDC conducts the national YRBS biennially and uses a three-stage cluster sample design to obtain nationally representative samples of students in grades 9–12 who attend public and private schools. In 2017, the school and student response rates were 75% and 81% respectively, resulting in an overall response rate of 60%.^¶^

State education and health agencies conduct state YRBSs and employ a two-stage cluster sample design to obtain state-representative samples of students in grades 9–12 who attend public schools. These samples are independent of the national YRBS. Among 46 states that administered YRBS in 2017, 33 states asked all six questions included in the national YRBS about fruits and vegetables, had sufficient response rates (>60%) to obtain weighted state-representative data, and gave CDC permission to include their data. State response rates ranged from 60% to 82%.

The six questions about fruit and vegetable consumption in the 2017 YRBS assess how many times per day or week students consumed 100% fruit juice, fruit, green salad, potatoes (excluding French fries, fried potatoes, and potato chips), carrots, and other vegetables.** The seven response options were 0, 1–3 or 4–6 times during the past 7 days; or 1, 2, 3, or 4 or more times daily. Daily frequency of fruit and vegetable intake was calculated by using the midpoint for intake ranges (e.g., five for 4–6 times during past 7 days) and dividing by seven for intakes reported by week. Student-reported race/ethnicity was classified into non-Hispanic White, non-Hispanic Black, Hispanic, and non-Hispanic other. Estimates for non-Hispanic other are not presented because this category includes multiple racial groups, which makes it difficult to provide meaningful interpretation, but are included in overall and other demographic estimates.

Among 14,765 students in the national YRBS, 1,411 (9.6%) were excluded, including 988 who did not answer fruit and vegetable questions; 376 who did not report sex, grade, or race/ethnicity; and 47 who were aged ≤14 years (to correspond to the age range used in the algorithm described in this report). The final analytic sample was 13,354 students. Similar exclusions were made on state-specific data with 5%–18% excluded across the states.

Median frequencies of fruit and vegetable intake were determined nationally and for 33 states. Previously established scoring algorithms (*2*), developed using 24-hour recall data from the National Health and Nutrition Examination Survey, were used to predict whether students met recommendations for their age and sex based on the number of times per day they reported consuming fruits and vegetables, separately, and accounting for race/ethnicity (*2*). Balanced repeated replication, replicate weights, and Taylor linearization were used to calculate 95% CIs for the percentage meeting recommendations (*2*). For vegetables, given CIs that include zero, only upper bounds are shown. T-tests were used to examine statistical differences by demographics (p-value <0.05). Analyses were performed using SAS (version 9.4; SAS Institute) survey procedures to account for complex sampling designs and Stata (version 14.0; StataCorp) to conduct t-tests.

Among students in grades 9–12 in 2017, the median reported daily intake was 0.9 times per day for fruits and 1.1 times per day for vegetables (Table 1). Nationally, 7.1% of high school students met federal intake recommendations for fruits (95% CI = 4.0–10.3), and 2.0% met these recommendations for vegetables (upper 95% CI = 7.9). State-specific estimates ranged from 4.0% (Connecticut) to 9.3% (Louisiana) for fruits and from 0.6% (Kansas) to 3.7% (New Mexico) for vegetables. Estimates were consistently low across demographic groups. Nationally, the percentage of students meeting recommendations for fruit consumption was higher among males (9.7%) than among females (4.7%), and higher among non-Hispanic Black persons (11.9%) and Hispanic persons (7.9%) than among non-Hispanic White persons (5.9%). These differences were not statistically significant, and patterns were similar in most states (Table 2). Similar, albeit less pronounced differences by sex and race/ethnicity were observed for the percentage of students meeting vegetable recommendations, nationally and in some states (Table 3). Estimates were similar across grade levels.

**TABLE 1:** Median consumption and percentages of high school students meeting U.S. Department of Agriculture fruit and vegetable* intake recommendations — Youth Risk Behavior Surveillance System, national and 33 states, 2017

State	No.^§^	Median times per day		Percentage meeting recommendations^†^	
				Fruit	Vegetable
		Fruit	Vegetable	% (95% CI)	% (Upper 95% CL)^¶^
**National**	**13,354**	**0.9**	**1.1**	**7.1 (4.0–10.3)**	**2.0 (7.9)**
Alaska	1,239	0.8	1.0	5.6 (1.9–9.3)	2.3 (8.8)
Arizona	1,936	0.8	1.0	4.8 (1.4–8.2)	1.2 (7.4)
Arkansas	1,376	0.7	0.9	7.6 (3.4–11.9)	3.6 (11.4)
California	1,640	0.9	1.0	6.1 (2.7–9.4)	1.8 (8.0)
Connecticut	2,109	0.9	1.2	4.0 (0.8–7.2)	1.0 (7.1)
Florida	5,644	0.9	1.0	7.4 (4.1–10.7)	2.2 (8.6)
Hawaii	5,174	0.7	1.0	4.4 (1.4–7.5)	1.7 (7.5)
Idaho	1,729	0.9	1.1	5.2 (1.7–8.7)	1.3 (7.4)
Illinois	4,331	0.8	1.0	5.4 (1.9–9.0)	1.3 (7.5)
Iowa	1,552	0.8	1.0	5.4 (1.5–9.2)	1.4 (7.7)
Kansas	2,288	0.9	1.1	4.2 (0.8–7.6)	0.6 (6.7)
Kentucky	1,867	0.7	0.9	5.0 (1.6–8.4)	1.0 (7.0)
Louisiana	1,070	0.7	0.8	9.3 (5.1–13.5)	1.9 (8.7)
Maryland	43,802	0.8	1.0	5.6 (2.7–8.4)	1.2 (7.2)
Massachusetts	2,998	0.9	1.1	5.2 (1.8–8.6)	1.1 (7.2)
Michigan	1,506	0.9	1.0	5.8 (1.8–9.7)	1.6 (8.0)
Missouri	1,648	0.7	1.0	4.8 (1.4–8.1)	1.0 (7.1)
Montana	4,475	0.8	1.1	4.6 (1.2–7.9)	1.6 (7.6)
Nebraska	1,306	0.8	1.1	4.5 (0.9–8.0)	1.0 (7.3)
New Mexico	5,333	0.8	1.1	7.5 (4.5–10.6)	3.7 (10.1)
North Carolina	2,837	0.8	1.0	6.1 (2.6–9.6)	1.2 (7.4)
North Dakota	2,009	0.9	1.1	5.3 (1.5–9.0)	1.4 (7.4)
Oklahoma	1,526	0.7	0.9	4.9 (1.6–8.3)	1.1 (7.2)
Pennsylvania	3,344	0.8	1.1	5.7 (2.2–9.1)	1.1 (7.2)
Rhode Island	2,017	0.8	1.0	5.9 (2.5–9.3)	2.2 (8.7)
South Carolina	1,310	0.7	0.9	7.0 (2.9–11.1)	0.9 (7.3)
Tennessee	1,894	0.8	0.9	6.3 (2.5–10.0)	1.6 (7.9)
Texas	1,955	0.8	0.9	6.0 (2.9–9.0)	1.6 (7.9)
Utah	1,712	0.8	1.1	4.6 (1.2–8.1)	1.7 (7.9)
Vermont	19,126	1.0	1.4	5.6 (2.4–8.9)	2.0 (7.9)
Virginia	3,431	0.8	1.1	5.5 (2.1–8.8)	1.7 (7.9)
West Virginia	1,414	0.8	1.0	6.8 (3.1–10.6)	1.5 (7.5)
Wisconsin	1,894	0.9	1.1	4.6 (1.0–8.3)	1.2 (7.5)

**Abbreviations:** CI = confidence interval; CL = confidence limit.

* Fruit consists of solid fruit and 100% fruit juice. Vegetable consists of green salad, potatoes (excluding French fries, fried potatoes, and potato chips), carrots, and other vegetables.

**^†^** Minimum daily intake recommendations for adolescents aged 14–18 years are: 1.5 cups of fruit and 2.5 cups of vegetables for females, and 2 cups of fruit and 3 cups of vegetables for males, per the U.S. Department of Agriculture. Additional information available at https://www.choosemyplate.gov/eathealthy/vegetables and https://www.choosemyplate.gov/eathealthy/fruits. Previously established scoring algorithms, developed using 24-hour recall data from the National Health and Nutritional Examination Survey, were used to predict whether students met recommendations for their age and sex based on the number of times per day they reported consuming fruits and vegetables, separately, and accounting for race/ethnicity.

^§^ Number of respondents aged 14–18 years with complete data for fruit and vegetable intake and demographic information.

^¶^ One-sided 95% CLs (i.e., upper bound only) are presented because of low prevalence of percentage meeting vegetable recommendations and CIs that include zero.

**TABLE 2:** National and state-specific percentages* and 95% confidence intervals (CIs) of high school students meeting U.S. Department of Agriculture fruit intake recommendations by sex, grade, and race/ethnicity — Youth Risk Behavior Surveillance System, national and 33 states, 2017

State	No.	% (95% CI)								
		Sex		Grade				Race/Ethnicity^†^		
		Female^§^	Male	9^§^	10	11	12	White^§^	Black	Hispanic
**National**	**13,354**	**4.7 (0.6–8.8)**	**9.7 (4.8–14.6)**	**7.6 (3.4–11.9)**	**7.6 (3.6–11.6)**	**6.7 (2.6–10.7)**	**6.4 (2.3–10.6)**	**5.9 (1.8–10.0)**	**11.9 (5.5–18.3)**	**7.9 (4.1–11.7)**
Alaska	1,239	3.6 (0.0^¶^–7.8)	7.5 (1.8–13.1)	6.5 (1.5–11.5)	5.8 (0.9–10.6)	4.9 (0.0–9.9)	5.1 (0.0–10.2)	4.1 (0.0–8.5)	—**	8.0 (1.3–14.7)
Arizona	1,936	2.3 (0.0–6.6)	7.3 (2.2–12.3)	6.0 (1.6–10.5)	5.0 (0.3–9.7)	4.0 (0.0–8.4)	4.1 (0.0–9.4)	4.0 (0.0–8.2)	—	5.2 (1.0–9.4)
Arkansas	1,376	5.2 (0.0–11.3)	10.0 (4.3–15.7)	7.9 (2.9–12.9)	6.7 (1.6–11.8)	6.3 (0.5–12.0)	9.7 (2.4–17.1)	6.2 (1.8–10.5)	11.8 (5.1–18.6)	5.2 (1.1–9.2)
California	1,640	3.6 (0.0–8.4)	8.6 (3.8–13.4)	5.8 (1.3–10.2)	6.0 (1.4–10.6)	6.9 (1.8–12.0)	5.7 (0.8–10.6)	5.3 (0.0–10.8)	5.8 (0.0–13.2)	6.9 (2.9–11.0)
Connecticut	2,109	2.2 (0.0–6.5)	5.8 (0.7–10.9)	4.4 (0.0–8.8)	3.7 (0.0–8.0)	4.3 (0.0–8.6)	3.6 (0.0–8.0)	3.2 (0.0–7.2)	5.1 (0.0–12.3)	5.3 (1.3–9.3)
Florida	5,644	5.2 (0.1–10.2)	9.6 (5.0–14.2)	7.8 (3.5–12.1)	7.4 (2.8–11.9)	8.1 (3.6–12.5)	6.3 (1.8–10.8)	5.1 (1.1–9.2)	9.8 (3.8–15.9)	8.2 (4.4–12.0)
Hawaii	5,174	2.8 (0.0–6.3)	6.2 (0.9–11.5)	5.2 (0.8–9.5)	4.0 (0.0–8.1)	4.1 (0.0–8.2)	4.4 (0.3–8.6)	4.3 (0.0–8.9)	—	5.2 (0.9–9.6)
Idaho	1,729	2.5 (0.0–6.3)	7.9 (2.0–13.8)	5.6 (1.0–10.3)	5.4 (1.1–9.7)	4.9 (0.1–9.6)	4.7 (0.0–9.6)	4.7 (0.4–9.1)	—	6.6 (2.1–11.2)
Illinois	4,331	3.8 (0.0–8.5)	7.1 (1.7–12.5)	5.1 (0.4–9.8)	4.9 (0.1–9.6)	5.5 (0.5–10.5)	6.3 (0.9–11.6)	4.2 (0.0–8.8)	7.4 (1.0–13.8)	6.7 (2.7–10.7)
Iowa	1,552	2.8 (0.0–7.1)	7.9 (1.4–14.3)	5.3 (0.1–10.5)	6.9 (1.3–12.5)	5.1 (0.0–10.4)	4.1 (0.0–9.6)	4.8 (0.5–9.2)	6.7 (0.0–15.6)	9.0 (1.1–16.9)
Kansas	2,288	2.4 (0.0–6.6)	5.9 (0.5–11.3)	4.8 (0.3–9.4)	3.4 (0.0–7.5)	4.0 (0.0–8.4)	4.6 (0.0–9.3)	3.8 (0.0–7.9)	4.7 (0.0–12.7)	4.9 (0.7–9.1)
Kentucky	1,867	2.9 (0.0–6.9)	7.1 (1.5–12.6)	5.0 (0.3–9.7)	4.4 (0.2–8.7)	6.6 (1.3–12)	4.0 (0.0–8.6)	4.8 (0.8–8.9)	5.2 (0.0–11.9)	7.6 (1.0–14.1)
Louisiana	1,070	7.6 (1.4–13.7)	11.1 (4.9–17.4)	8.9 (3.0–14.8)	8.0 (1.7–14.2)	9.1 (1.7–16.6)	11.5 (4.0–19.0)	7.3 (2.2–12.5)	11.6 (4.3–19.0)	—
Maryland	43,802	3.7 (0.0–7.9)	7.5 (3.2–11.7)	5.5 (1.8–9.3)	5.8 (1.9–9.6)	5.5 (1.7–9.3)	5.3 (1.5–9.2)	4.3 (0.7–7.8)	7.3 (2.4–12.1)	6.2 (3.0–9.4)
Massachusetts	2,998	3.1 (0.0–7.2)	7.4 (2.0–12.7)	4.4 (0.1–8.7)	6.4 (1.6–11.3)	5.0 (0.1–10.0)	5.0 (0.6–9.3)	4.3 (0.0–8.5)	10.2 (3.6–16.8)	5.8 (1.9–9.7)
Michigan	1,506	3.2 (0.0–7.8)	8.3 (2.1–14.4)	5.2 (0.2–10.1)	6.9 (1.1–12.7)	5.4 (0.2–10.6)	5.6 (0.0–11.2)	4.7 (0.5–8.9)	10.5 (2.9–18.1)	8.6 (3.2–14.0)
Missouri	1,648	2.1 (0.0–6.1)	7.5 (2.1–12.9)	4.3 (0.0–8.8)	6.1 (1.3–11.0)	4.7 (0.0–9.6)	3.8 (0.0–8.5)	4.3 (0.5–8.2)	5.6 (0.0–12.3)	6.8 (1.6–11.9)
Montana	4,475	2.5 (0.0–6.0)	6.5 (0.8–12.2)	5.0 (0.7–9.4)	4.9 (0.7–9.1)	4.1 (0.0–8.4)	4.1 (0.0–8.4)	4.1 (0.3–7.9)	—	7.9 (3.3–12.5)
Nebraska	1,306	2.2 (0.0–6.5)	6.7 (1.0–12.4)	6.2 (1.0–11.4)	3.9 (0.0–9.1)	4.6 (0.0–9.5)	3.1 (0.0–7.9)	4.0 (0.0–8.2)	—	6.0 (0.5–11.5)
New Mexico	5,333	5.3 (0.5–10.2)	9.7 (5.6–13.8)	7.7 (3.6–11.9)	7.2 (3.1–11.3)	7.5 (3.4–11.5)	7.6 (3.3–12.0)	5.8 (1.5–10.2)	—	7.5 (3.9–11.0)
North Carolina	2,837	4.0 (0.0–9.0)	8.2 (3.1–13.4)	5.4 (0.9–9.9)	5.6 (1.2–10.1)	7.6 (2.2–13.0)	5.8 (1.1–10.5)	4.3 (0.1–8.5)	8.9 (2.0–15.8)	7.2 (2.1–12.3)
North Dakota	2,009	2.6 (0.0–6.4)	7.8 (1.5–14.2)	5.4 (0.6–10.1)	4.9 (0.2–9.6)	5.5 (0.0–11.4)	5.4 (0.3–10.5)	4.6 (0.3–9.0)	—	8.1 (2.1–14.0)
Oklahoma	1,526	2.2 (0.0–6.0)	7.8 (2.2–13.3)	3.6 (0.0–7.6)	7.3 (2.1–12.5)	5.1 (0.2–10.1)	3.6 (0.0–8.5)	4.7 (0.3–9.1)	—	6.1 (1.7–10.4)
Pennsylvania	3,344	3.3 (0.0–7.4)	8.0 (2.4–13.6)	6.3 (1.4–11.2)	6.1 (1.3–10.9)	5.2 (0.5–9.9)	5.0 (0.5–9.6)	4.5 (0.4–8.5)	9.7 (2.8–16.6)**^††^**	11.1 (6.0–16.1)
Rhode Island	2,017	3.0 (0.0–7.4)	8.6 (3.0–14.2)	6.7 (1.7–11.7)	7.1 (1.8–12.3)	5.3 (0.3–10.3)	4.1 (0.0–8.8)	5.0 (0.6–9.4)	8.5 (0.9–16.1)	6.9 (2.4–11.4)
South Carolina	1,310	6.1 (0.0–12.1)	8.0 (2.5–13.6)	8.9 (2.9–14.9)	7.2 (1.9–12.5)	5.7 (0.0–11.5)	6.0 (0.0–12.4)	4.5 (0.1–9.0)	10.7 (3.6–17.8)	8.8 (1.3–16.3)
Tennessee	1,894	4.3 (0.0–9.0)	8.2 (2.3–14.0)	6.3 (1.1–11.6)	6.2 (1.3–11.1)	7.0 (1.8–12.1)	5.5 (0.5–10.6)	5.5 (1.0–10.1)	8.0 (1.8–14.3)	6.7 (1.6–11.8)
Texas	1,955	4.6 (0.0–9.7)	7.3 (3.1–11.6)	7.8 (3.4–12.1)	4.8 (0.4–9.2)	5.0 (0.0–10.0)	6.1 (1.0–11.2)	4.4 (0.0–9.0)	9.7 (2.2–17.3)	5.8 (2.3–9.3)
Utah	1,712	2.1 (0.0–5.9)	7.1 (1.3–12.9)	5.2 (0.3–10.2)	4.8 (0.1–9.5)	4.3 (0.0–8.8)	4.1 (0.0–8.9)	4.1 (0.0–8.3)	—	6.5 (1.8–11.2)
Vermont	19,126	2.7 (0.0–6.1)	8.4 (2.9–13.9)	5.6 (1.6–9.7)	5.7 (1.7–9.8)	5.5 (1.5–9.5)	5.6 (1.6–9.6)	5.2 (1.4–9.0)	10.5 (4.3–16.6)	8.4 (4.1–12.7)
Virginia	3,431	3.2 (0.0–7.8)	7.6 (2.7–12.6)	6.1 (1.5–10.8)	5.1 (0.6–9.5)	5.7 (0.9–10.6)	4.9 (0.2–9.5)	4.0 (0.1–8.0)	8.0 (1.5–14.4)	6.7 (2.3–11.0)
West Virginia	1,414	4.6 (0.2–8.9)	9.1 (2.8–15.3)	7.5 (2.0–13.0)	7.8 (2.1–13.4)	6.7 (1.7–11.6)	5.3 (0.2–10.4)	6.5 (2.3–10.8)	—	—
Wisconsin	1,894	1.8 (0.0–5.7)	7.4 (1.5–13.4)	4.6 (0.0–9.4)	6.2 (1.3–11.1)	3.9 (0.0–8.6)	3.9 (0.0–8.4)	4.3 (0.0–8.6)	6.8 (0.1–13.5)	5.2 (0.7–9.6)

* National data are weighted and representative of all private and public school students in grades 9–12 in the United States. State data are weighted and representative of all public school students in grades 9–12 in the respective jurisdiction.

^†^ White and Black students are non-Hispanic. Hispanic students could be of any race. Non-Hispanic other group not reported because this group includes multiple racial groups, which makes it difficult to provide meaningful interpretation, but included in overall estimates and estimates by other demographic characteristics.

^§^ Female sex, grade 9, and White race/ethnicity were used as referents.

^¶^ Negative values for lower CI bounds were truncated at zero.

** Dashes indicate that estimates in states where the sample size was <100 were considered unstable and were not reported.

**^††^** P<0.05 for t-test comparing differences by demographic groups to the referent group.

**TABLE 3:** National and state-specific percentages* and one-sided 95% upper confidence limits (CLs) of high school students meeting U.S. Department of Agriculture vegetable intake recommendations by sex, grade, and race/ethnicity **—** Youth Risk Behavior Surveillance System, national and 33 states, 2017

State	No.	% (95% CL)^†^								
		Sex		Grade				Race/Ethnicity^§^		
		Female^¶^	Male	9^¶^	10	11	12	White^¶^	Black	Hispanic
**National**	**13,354**	**1.1 (2.8)**	**3.0 (14.8)**	**2.2 (8.3)**	**2.4 (8.5)**	**1.5 (7.5)**	**2.0 (8.2)**	**1.7 (7.4)**	**2.2 (10.1)**	**2.6 (9.1)**
Alaska	1,239	0.9 (3.1)	3.7 (16.0)	2.1 (9.1)	2.9 (10.3)	1.3 (8.3)	3.0 (10.6)	1.4 (8.0)	—**	4.1 (13.9)
Arizona	1,936	0.6 (1.8)	1.9 (13.8)	2.0 (8.5)	0.9 (7.3)	0.6 (6.8)	1.4 (8.2)	1.0 (7.3)	—	1.7 (8.2)
Arkansas	1,376	2.6 (7.9)	4.6 (18.4)	3.4 (11.3)	3.0 (10.7)	1.6 (8.2)	6.7 (19.9)	3.0 (11.1)	5.4 (15.6)	3.8 (13.9)
California	1,640	0.8 (2.2)	2.7 (15.1)	2.1 (8.8)	1.5 (8.3)	2.5 (9.4)	1.0 (7.8)	0.2 (5.7)	0.0 (7.5)	3.0 (9.8)
Connecticut	2,109	0.6 (2.0)	1.4 (13.4)	1.7 (8.3)	1.0 (7.4)	0.9 (7.1)	0.4 (6.7)	1.0 (7.2)	0.7 (7.8)	1.2 (8.2)
Florida	5,644	1.0 (2.8)	3.4 (15.8)	2.1 (8.6)	2.6 (9.6)	2.0 (8.7)	2.2 (8.9)	2.3 (8.6)	1.5 (9.4)	2.6 (8.7)
Hawaii	5,174	0.9 (2.7)	2.6 (14.4)	1.9 (8.2)	1.8 (8.1)	1.4 (7.5)	1.7 (7.9)	2.1 (9.4)	—	1.6 (7.7)
Idaho	1,729	0.7 (2.5)	1.9 (13.8)	1.6 (8.1)	0.9 (7.4)	1.4 (7.9)	1.3 (7.9)	1.0 (7.0)	—	2.4 (9.3)
Illinois	4,331	0.6 (2.0)	2.0 (14.3)	1.2 (7.5)	1.2 (7.6)	0.8 (7.2)	2.0 (9.1)	1.0 (7.1)	0.9 (8.3)	2.0 (9.2)
Iowa	1,552	0.9 (2.4)	2.0 (14.1)	1.3 (8.1)	3.3 (11.5)	0.9 (7.2)	0.1 (6.4)	1.3 (7.4)	2.0 (12.4)	1.4 (10.6)
Kansas	2,288	0.4 (1.5)	0.8 (12.6)	0.5 (6.8)	0.3 (6.4)	0.8 (7.4)	0.8 (7.3)	0.6 (6.7)	0.3 (7.4)	0.7 (7.1)
Kentucky	1,867	0.4 (1.4)	1.6 (13.5)	1.5 (8.1)	0.4 (6.5)	1.2 (7.7)	1.0 (7.5)	1.0 (7.0)	0.1 (7.9)	2.4 (11.7)
Louisiana	1,070	0.6 (2.0)	3.2 (17.2)	2.1 (9.9)	2.0 (9.0)	1.4 (8.4)	2.1 (11.5)	2.5 (9.9)	1.2 (8.6)	—
Maryland	43,802	0.6 (1.6)	1.9 (14.0)	1.4 (7.6)	1.1 (7.2)	1.3 (7.4)	1.2 (7.2)	1.1 (6.7)	1.1 (8.2)	2.0 (8.3)
Massachusetts	2,998	0.6 (1.9)	1.7 (13.6)	1.0 (7.4)	1.3 (7.8)	1.5 (8.3)	0.8 (6.9)	0.8 (6.7)	0.8 (9.2)	2.0 (8.6)
Michigan	1,506	0.4 (1.8)	2.7 (15.2)	1.6 (8.2)	1.5 (8.5)	1.7 (9.0)	1.5 (8.5)	1.3 (7.5)	1.6 (11.2)	3.7 (11.9)
Missouri	1,648	0.2 (1.0)	1.9 (14.0)	1.4 (8.1)	1.3 (7.9)	0.6 (6.6)	0.8 (7.1)	1.0 (6.7)	0.0 (8.2)	3.1 (11.8)
Montana	4,475	0.7 (2.4)	2.3 (13.9)	1.4 (7.8)	1.2 (7.6)	1.6 (8.0)	2.0 (8.4)	1.3 (7.3)	—	3.6 (11.6)
Nebraska	1,306	0.4 (1.5)	1.6 (14.0)	0.4 (6.7)	1.4 (8.1)	0.6 (7.0)	1.7 (9.4)	0.8 (7.0)	—	0.4 (7.2)
New Mexico	5,333	1.7 (4.0)	5.7 (18.1)	4.3 (11.2)	2.6 (9.2)	4.3 (11.4)	3.7 (10.4)	2.9 (9.9)	—	4.0 (10.5)
North Carolina	2,837	0.8 (2.1)	1.7 (14.0)	1.2 (7.8)	1.1 (7.4)	1.1 (7.8)	1.5 (8.0)	1.1 (7.2)	1.0 (8.5)	2.1 (8.9)
North Dakota	2,009	0.6 (2.4)	2.1 (13.9)	1.1 (7.5)	1.5 (7.9)	1.4 (7.8)	1.5 (8.4)	1.3 (7.3)	—	3.9 (12.7)
Oklahoma	1,526	0.6 (2.1)	1.7 (13.6)	0.8 (6.9)	2.0 (8.7)	0.9 (7.4)	0.8 (7.7)	1.0 (7.2)	—	1.6 (8.5)
Pennsylvania	3,344	0.6 (1.7)	1.7 (13.6)	2.1 (8.8)	1.0 (7.5)	0.8 (7.2)	0.7 (7.0)	1.0 (7.0)	1.0 (9.6)	3.3 (10.6)
Rhode Island	2,017	0.8 (2.5)	3.6 (15.8)	2.5 (9.7)	2.8 (10.0)	1.8 (8.4)	1.7 (8.8)	1.9 (8.3)	2.2 (12.8)	2.7 (10.3)
South Carolina	1,310	0.8 (2.7)	1.1 (13.6)	1.2 (8.4)	0.8 (7.7)	1.1 (7.5)	0.5 (7.3)	1.1 (7.6)	0.5 (7.6)	1.6 (9.2)
Tennessee	1,894	0.7 (2.1)	2.6 (14.9)	1.5 (8.6)	1.5 (8.5)	1.3 (7.8)	2.3 (9.6)	1.4 (7.5)	1.8 (10.4)	2.7 (10.2)
Texas	1,955	0.5 (1.9)	2.7 (15.1)	1.8 (8.7)	0.6 (7.3)	1.1 (7.7)	2.9 (10.4)	0.6 (6.5)	3.6 (13.9)	1.6 (7.9)
Utah	1,712	0.7 (2.7)	2.7 (14.6)	1.4 (7.7)	1.7 (8.5)	1.4 (8.2)	2.5 (,9.3)	1.4 (7.7)	—	3.2 (10.6)
Vermont	19,126	1.0 (2.8)	2.9 (14.4)	1.9 (7.9)	2.1 (8.1)	1.7 (7.9)	2.1 (8.3)	1.7 (7.5)	3.8 (13.8)	4.9 (12.8)
Virginia	3,431	0.7 (2.2)	2.7 (14.9)	2.2 (9.0)	1.5 (8.0)	1.5 (8.2)	1.8 (8.4)	1.4 (7.6)	1.1 (8.7)	3.7 (11.7)
West Virginia	1,414	0.8 (2.2)	2.2 (13.9)	0.8 (7.2)	2.6 (9.8)	2.0 (8.5)	0.5 (6.5)	1.6 (7.5)	—	—
Wisconsin	1,894	0.6 (2.0)	1.9 (14.2)	1.0 (7.6)	1.4 (8.4)	1.3 (8.0)	1.2 (8.1)	1.2 (7.3)	1.7 (11.5)	1.2 (9.3)

* National data are weighted and representative of all private and public school students in grades 9–12 in the United States. State data are weighted and representative of all public school students in grades 9–12 in the respective jurisdiction.

^†^ One-sided 95% CLs (i.e., upper bound only) are presented because of low prevalence of percentage meeting recommendations and CIs that include zero.

^§^ White and Black students are non-Hispanic. Hispanic students could be of any race. Non-Hispanic other group not reported because this group includes multiple racial groups, which makes it difficult to provide meaningful interpretation, but included in overall estimates and estimates by other demographic characteristics.

^¶^ Female sex, grade 9, and White race/ethnicity were used as referents.

** Dashes indicate that estimates in states where the sample sizes were <100 were considered unstable and were not reported.

## Discussion

The proportion of U.S. high school students meeting federal intake recommendations remained low in 2017, with 7.1% consuming enough fruits and 2.0% consuming enough vegetables to meet USDA recommendations. Although estimating the tail ends of distributions can be less precise than estimating mean intake (2), results still indicate that consumption across all demographic groups was insufficient to meet dietary recommendations. These findings are consistent with other studies indicating that adolescents consume fruits and vegetables much less frequently than is recommended for proper nutrition (*2*–*4*).

Reasons for insufficient consumption of fruits and vegetables by adolescents are complex. Adolescents might face barriers to consumption, including high availability of inexpensive, unhealthy food options, lack of taste preference for fruits and vegetables, and lack of home availability (*5*). Interventions to address some of these barriers are occurring in schools and community programs. For example, the National School Lunch Program^††^ requires that meals include a fruit and vegetable option daily. However, on average, 39% of high school students participate in the National School Lunch Program (*6*), and fewer (14%) participate in the School Breakfast Program; participation is particularly low among students who do not qualify for free or reduced-price meals. Smart Snacks Standards ensure that foods and beverages sold in vending machines, school stores, and fundraisers include nutritious options, including fruits and vegetables (*7*). In addition, state and local farm-to-school programs support experiential learning activities, including cooking and taste-testing, to engage students in preparing and eating fruits and vegetables.^§§^

Community programs can also reduce barriers to fruit and vegetable consumption, including lack of home availability of fruits and vegetables, which is a consistent correlate of intake among adolescents (*8*). For example, projects funded by the Gus Schumacher Nutrition Incentive Program^¶¶^ support families with low income by providing financial incentives to purchase more produce. Additional communication approaches, including parent-directed messaging about exposing children to nutritious foods early and repeatedly at home,*** might enhance preferences for fruits and vegetables. Further, social marketing and health-branding strategies, such as those used by the FNV Campaign,^†††^ to positively influence attitudes and societal norms about eating fruits and vegetables, might appeal to adolescents, particularly those who use social media. Consistently low fruit and vegetable intake among adolescents suggests that additional efforts are needed to expand the reach of existing programs or to identify new effective strategies such as communication approaches including social media.

The findings in this report are subject to at least two limitations. First, YRBS data are self-reported, which might overestimate fruit and vegetable intake (*9*). Second, intake recommendations are based on adolescents who do not engage in ≥30 minutes of physical activity daily, and active persons should consume more. These results might overestimate percentages meeting recommendations because 46.5% of U.S. students were active for ≥60 minutes per day on 5 or more days.^§§§^

YRBS data are highly representative of the adolescent population because 96% of adolescents aged 14–17 years attend school (*10*). Despite the benefits of healthy eating, these findings indicate that most high school students do not consume enough fruits and vegetables to meet USDA recommendations. Continued efforts to identify and address barriers to consumption might help adolescents eat more fruits and vegetables and support their overall health.

**Summary**

**What is already known about this topic?**

Consuming fruits and vegetables is associated with reduced risk for cardiovascular disease, type 2 diabetes, some cancers, and obesity. Healthy dietary habits established during adolescence might continue into adulthood. However, few U.S. high-school students eat enough fruits and vegetables to meet U.S. Department of Agriculture recommendations.

**What is added by this report?**

Adolescents in all demographic groups consume too few fruits and vegetables, consistent with previous findings. In 2017, 7.1% met fruit intake recommendations, and 2.0% met vegetable intake recommendations.

**What are the implications for practice?**

Efforts to expand the reach of existing school and community programs as well as identify new strategies, such as social media approaches, might be needed to increase fruit and vegetable consumption among adolescents.

## Footnotes

* https://www.dietaryguidelines.gov/sites/default/files/2020-12/Dietary_Guidelines_for_Americans_2020-2025.pdf.

† These amounts are appropriate for persons who get <30 minutes per day of moderate physical activity, beyond normal daily activities. Those who are more physically active might be able to consume more while staying within calorie needs. https://www.choosemyplate.gov/eathealthy/vegetables, https://www.choosemyplate.gov/eathealthy/fruits.

§ Methodology of the Youth Risk Behavior Surveillance System–2013 https://www.cdc.gov/mmwr/pdf/rr/rr6201.pdf.

¶ https://www.cdc.gov/healthyyouth/data/yrbs/pdf/2017/ss6708.pdf.

** https://www.cdc.gov/healthyyouth/data/yrbs/pdf/2017/2017_yrbs_national_hs_questionnaire.pdf.

†† https://www.fns.usda.gov/nslp.

§§ https://www.fns.usda.gov/cfs/farm-school-grant-program.

¶¶ https://nifa.usda.gov/program/gus-schumacher-nutrition-incentive-grant-program.

*** https://www.healthychildren.org/English/healthy-living/growing-healthy/Pages/preschool-food-and-feeding.aspx#none.

††† https://fnv.com/about/.

§§§ https://www.cdc.gov/healthyyouth/data/yrbs/pdf/2017/ss6708.pdf.
